# A Comprehensive Survey of Depth Completion Approaches

**DOI:** 10.3390/s22186969

**Published:** 2022-09-14

**Authors:** Muhammad Ahmed Ullah Khan, Danish Nazir, Alain Pagani, Hamam Mokayed, Marcus Liwicki, Didier Stricker, Muhammad Zeshan Afzal

**Affiliations:** 1 Department of Computer Science, Technical University of Kaiserslautern, 67663 Kaiserslautern, Germany; 2 Mindgarage, Technical University of Kaiserslautern, 67663 Kaiserslautern, Germany; 3 German Research Institute for Artificial Intelligence (DFKI), 67663 Kaiserslautern, Germany; 4 Department of Computer Science, Luleå University of Technology, 971 87 Luleå, Sweden

**Keywords:** depth completion, depth maps, image-guidance

## Abstract

Depth maps produced by LiDAR-based approaches are sparse. Even high-end LiDAR sensors produce highly sparse depth maps, which are also noisy around the object boundaries. Depth completion is the task of generating a dense depth map from a sparse depth map. While the earlier approaches focused on directly completing this sparsity from the sparse depth maps, modern techniques use RGB images as a guidance tool to resolve this problem. Whilst many others rely on affinity matrices for depth completion. Based on these approaches, we have divided the literature into two major categories; unguided methods and image-guided methods. The latter is further subdivided into multi-branch and spatial propagation networks. The multi-branch networks further have a sub-category named image-guided filtering. In this paper, for the first time ever we present a comprehensive survey of depth completion methods. We present a novel taxonomy of depth completion approaches, review in detail different state-of-the-art techniques within each category for depth completion of LiDAR data, and provide quantitative results for the approaches on KITTI and NYUv2 depth completion benchmark datasets.

## 1. Introduction

**Figure 1:**
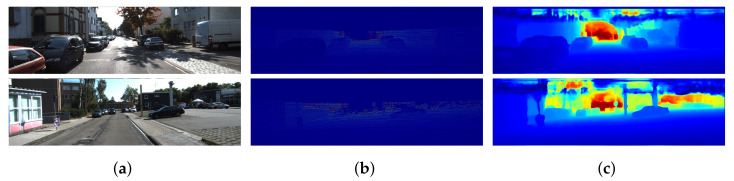
First Column shows the RGB images from two different scenes, the middle column contains the sparse depth maps produced from LiDAR. The last column shows the predicted dense depth maps for the corresponding scenes. (**a**) RGB Image. (**b**) LiDAR sparse Depth Map. (**c**) Prediction.

Depth maps are critical to a variety of computer vision applications such as autonomous driving [1,2,3], robot navigation [4,5], augmented reality [6,7,8], virtual reality [9]. Tasks like object detection, obstacle avoidance [10], 3D scene reconstruction [11,12,13] require dense depth maps for accurate prediction. Various depth sensors like depth cameras, 3D LiDAR, and stereo cameras capture the depth information. Among these, LiDAR sensors provide the most accurate depth information. However, the depth maps generated by these devices are sparsely distributed (Figure 1) compared to a medium resolution RGB image (about 5% density [14]). Also, current LiDAR sensors obtain measurements at only 64 scan lines in the vertical direction. This sparsity significantly impacts the performance of LiDAR-based applications. Predicting dense depth maps from these sparse ones is critical for both the industry and academia.

To resolve the problem of depth completion, many different approaches have been developed. Approaches like [15,16,17] concentrate on retrieving dense depth maps from the sparse ones without the guidance of an image. Uhrig et al. [18] propose a sparsity invariant CNN to handle the sparsity in LiDAR data and its corresponding features. Eldesokey et al. [19] introduce normalized convolutional layer for unguided scene depth completion by using confidence propagations. But, these approaches are limited and lose depth of details and semantic information without the availability of multi-modal data.

Image-guided methods show significant improvement in results compared to the conventional depth-only techniques. Qiu et al. [20] use deep learning for image-guided depth completion using surface normals. CSPN [21] extends the SPN to predict affinity matrices using CNN for depth completion. CSPN++ [22] further improves the CSPN approach by learning additional hyperparameters of convolution kernel sizes and the number of iterations for propagation, both are adaptive and content dependent. However, most of these techniques consider the task as one-stage learning and use naıve fusion approaches resulting in blurred depth maps with unclear boundaries.

Some works construct a multi-branch architecture for handling image and depth modalities and then perform fusion like FusionNet [23] and DeepLiDAR [20]. FusionNet extracts local and global features using its two-branch architecture. While, DeepLiDAR takes multi-modal inputs and performs fusion at a multi-scale level, achieving better depth completion results. But both these methods require extra datasets to pre-train their networks.

The content of this paper is organized as follows: Section 2 provides an overview of the fusion strategies and approaches used in the field of depth completion. Section 3 describes the fusion approaches in the literature. Section 4 discusses the common indoor and outdoor dataset used for depth completion. Section 5 introduces the metrics used in the field of depth completion. Section 6 describes the objective functions used in the literature and Section 7 presents the state-of-the-art methods in each category. Finally, Section 8 provides the conclusion of this paper.

## 2. Methodologies

**Figure 2:**
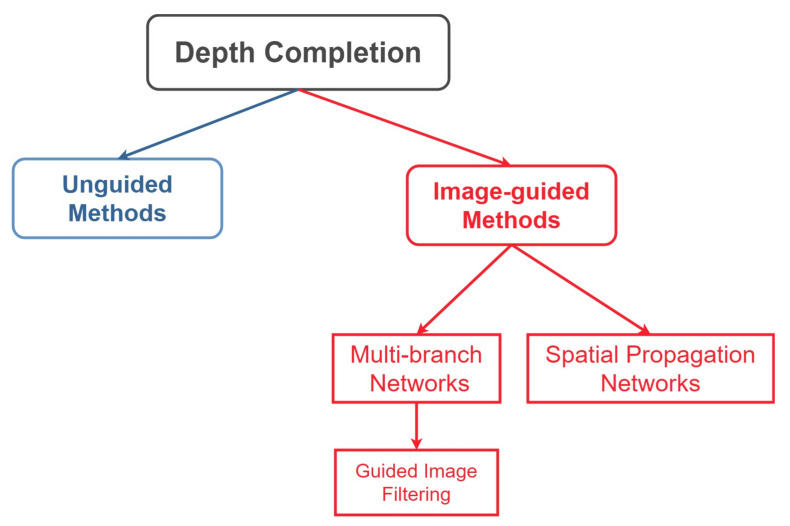
Approaches to depth completion problem. Unguided approaches utilize either only LiDAR information or confidence maps and LiDAR information for dense depth completion. The image-guided methods (multi-branch and spatial propagation networks) employ guidance images (RGB, semantic maps, surface normals) to guide the process of depth completion. The multi-branch networks can be further divided into guided image filtering methods, which aim to learn useful kernels from one modality and apply it to other modalities.

In this section, we will present the approaches to dense depth completion. Figure 2 shows the approaches to depth completion. Roughly, the approaches can be divided into two different categories; (1) Unguided methods, which utilize only LiDAR sparse depth maps for dense depth completion, and (2) Image-guided methods, which employ guidance images (RGB, semantic maps, surface normals) to guide the process of depth completion. Image-guided methods are more successful than unguided approaches. However, image-guided methods require the employment of fusion strategies to adaptively fuse the information between different modalities. Therefore, we also discuss multi-modal fusion strategies in Section 3.

### 2.1. Unguided Methods

Most of the earlier approaches [16,18,20] to depth completion employed only a single modality i.e., LiDAR sparse depth maps to generate dense depth maps. However, raw LiDAR sparse depth maps contain missing values at most of the pixels. To fill out the missing values at invalid regions of sparse depth maps, many hand-crafted features, kernels, interpolation methods [24,25,26,27,28] were introduced. However, the structural information of the scene is lost because of the discontinuity in the depth values. To enable learning from the convolutions, Depth-Net [29] first applied nearest-neighbor interpolation in the sparse maps to fill invalid depth values and then passed it to the deep neural network for learning.

As the field progressed, the idea of embedding auxiliary information such as confidence maps, etc., to enhance the quality of depth completion [30,31] gained more attention. Specifically, in the initial stage, confidence maps are generated. Later on, the LiDAR sparse depth maps along with confidence maps are taken as an input and passed to a deep neural network to complete the sparse depth maps. In [31], the confidence maps are generated from the convolution operation, whereas in uncertainty-aware CNN’s [30], they are generated on the base of self-supervision methodologies. These approaches achieved much better results than before. However, the predicted depth maps still lack clear structure, e.g., object boundaries. Thus, unsuitable for real-time applications.

### 2.2. Image-Guided Methods

Image-guided techniques refer to the ones that employ guidance images such as RGB images [32,33], semantic maps [34,35], surface normals [20] and sparse depth map modalities [18] to guide the process of depth completion. These techniques have shown much more efficient results compared to the unguided approaches.

#### 2.2.1. Multi-Branch Networks

Multi-branch networks refer to the ones that employ two or more branches for handling multi-modal information, including RGB images, surface normals, semantic maps, and LiDAR sparse depth maps. Each branch treats a single modality separately and then the information from the different branches is fused through multi-modal fusion techniques explained in Section 3.

Van Gansbeke, Wouter, et al. [23] propose a two-branch network to extract both the global and local information to produce accurate and comprehensive depth maps. They employ a fusion method based on color image guidance to better incorporate the object information, which significantly improves accuracy. Additionally, the depth maps from the two branches are weighted by their respective confidence masks, learned from unsupervised learning, to correct the uncertainty in depth.

DeepLiDAR [20] presents a deep learning architecture for accurate image-guided depth completion for outdoor scenes using estimated surface normals [36] as intermediate representations to enforce geometric constraints. The sparse depth and image modalities are effectively fused together by the proposed modified two-branch encoder-decoder network [37]. To resolve the issues specific to outdoor scenes, attention maps and confidence masks are used to improve the depth of distant objects and handle occlusions around object boundaries respectively.

Similar to DeepLiDAR [20], to resolve the issues in handling sensor noise and 3D geometric constraints, Xu et al. [38] propose a CNN framework with a prediction and a refinement module. The prediction module predicts a depth map along with its corresponding confidence map and surface normal [36] using an encoder-decoder network [37]. The confidence maps reduce the propagation of noise from LiDAR data. These predictions are then fused in a refinement module by mapping constraints from depth to surface normals.

Unlike the conventional approaches, which make a point estimate, Yang et al. [39] propose a system, which takes an image and a sparse aligned point cloud to predict a posterior probability over the depth values corresponding to each pixel in the scene. With the help of a Conditional Prior Network, the method finds relations between seen images and corresponding depth maps to get a probability at each depth value. Further using sparse measurements, it combines this probability with a likelihood term.

Ma et al. [15] design a deep learning regression model to directly predict the dense depth map from a sparse depth map and a color image (if available). To remove the requirement of dense depth labeling during the training cycle, the paper further proposes a self-supervised learning method that only takes sequences of sparse depth maps and color images. This approach performs better than even some of the semi-dense annotation methods.

The standard convolutions fail to model the observed spatial contexts due to sparsity in depth maps. To fully capture the observed spatial contexts, Zhao et al. [40] propose graph propagations. Multi-scale features are extracted by applying these propagations on multiple graphs obtained from observed pixels. Then an attention mechanism is applied to the propagation, which allows the modeling of the contextual information adaptively. These graph propagations are applied to the depth and image modalities to extract the respective representations. To comprehensively fuse the multi-modal features a fusion strategy is proposed which uses an adaptive gating mechanism and preserves the unique information of each modality while fusing them.

Li et al. extends hourglass [41] to a multi-scale guided cascade network for handling diverse patterns in depth maps efficiently [42]. Unlike the traditional fully convolutional techniques, the cascade network takes inputs at different resolutions to predict depth structures at particular scales. The network performs multi-level image guidance at different hourglasses. The division into sub-modules allows replacing the redundant network with a combination of simple architectures.

DenseLiDAR [43] propose a real-time pseudo-depth guided depth completion backbone based neural network. The authors argue that an intermediate dense depth map is much better to produce accurate dense predictions than a sparse map. The pseudo-depth map helps in predicting the residual depth providing better predictions. It further allows us to reduce the points in sparse depth causing an error. Additionally, two new metrics; $RMSE_{GT+}$ and $RMSE_{Edge}$ are proposed for depicting the true nature of predictions and better evaluation of depth completion tasks.

Most of the earlier mentioned image-guided depth completion methods use simple concatenation and element-wise addition to handle multi-modal fusion. The deep convolutional encoder-decoder architecture [37] designed by Lee et al. [44] incorporates a cross-guidance module for fusing the features from different modalities. The encoders from both stages share the information by exchanging the outputs with the guidance module of the other encoder, which applies an attention mechanism to fuse the features. To extract important features, a residual atrous spatial pyramid block (RASP) applies dilated convolutions [45] with non-similar dilation rates in parallel.

Inspired by the Sparsity Invariant Convolution (SI-Conv), proposed by Uhrig et al. [18] for depth-only completion tasks, Yan et al. [46] propose an image-guided deep learning approach for depth completion. It further presents a novel multi-modal fusion technique to effectively fuse the image and depth data. The main core of this approach are the three Mask Aware operations; Bottleneck, Pooling, and Fusion, which work together to process, downscale, and fuse the sparse data. The proposed novel fusion scheme makes use of a spatial pyramid block to fuse the features at multiple scales.

Different from previously discussed approaches which use a typical Convolution Neural Network (CNN) layer, the approach in [19] introduces a novel normalized convolutional layer with a much smaller number of parameters for unguided scenes depth completion on the highly sparse input depth map. It further presents novel methodologies to compute and propagate convolutional confidences to consequent CNN layers. A new loss function is also proposed, minimizing the data error while maximizing the output confidence. The authors also explore several fusion techniques to combine the multi-modal data and integrate structural information in the proposed framework. Additionally, unlike [15] the output confidence is used as auxiliary information to improve the results.

Sparse Spatial Guided Propagation (SSGP) [47] uses content-dependent and sparsity-aware convolutional propagations to interpolate sparse scenes, providing image guidance at all stages of the network. The encoder-decoder network performs sparse-to-dense interpolation for different problems like optical flow, scene flow, depth completion, etc., achieving better robustness, accuracy, and speed.

Contrary to the common depth completion approaches, FCFR-Net [48] treats the depth completion problem as a two-stage problem. In the first sparse-to-dense stage, a simple CNN [15] is used to interpolate the original sparse map to a coarse depth map. This coarse depth map is then refined in the second coarse-to-fine stage to get the final dense depth map. The coarse-to-fine stage employs a channel shuffle extraction operation and an energy fusion operation to extract discriminative and comprehensive features from both modalities and then fuse them together in a sufficient manner. The complete approach works as a residual learning framework.

Inspired by FusionNet [23] and DeepLiDAR [20], Hu et al. [32] propose a two-branch network PENet, consisting of a color dominant branch and a depth dominant branch. However, the branches are for different purposes and unlike [20,23], the network can be trained from scratch without requiring any additional datasets. Both branches focus on extracting the dominant and discriminative features from the corresponding modalities to generate dense depth maps. The two maps are then fused together with their confidence. Geometric constraints are also enforced through a geometric convolutional layer [49]. Finally, the fused maps are refined using a scheme based on CSPN++ [22], which implements dilated and accelerated propagations.

Motivated by the popular mechanism of looking and thinking twice in [50], RigNet [33] employs a repetitive design in the image-guided network and depth generation branch to gradually and sufficiently recover depth values, resolving the issues related to blurry image guidance and unclear structure in depth. The image guidance branch uses a repetitive hourglass network to produce multi-scale features with improved image semantics. The depth branch employs a repetitive guidance module consisting of dynamic convolutions [51]. This module has an adaptive fusion mechanism to aggregate the features and an efficient guidance algorithm to reduce the runtime caused by dynamic convolutions.

##### Guided Image Filtering

Guided Image Filtering is considered another variant of multi-branch methods. In the field of depth completion, the idea of guided image filtering refers to the learning and prediction of the kernels from one modality and applying learned kernels to other modalities for feature extraction and fusion.

This approach was first introduced by GuideNet [51]. It proposed a novel method for learning guided kernels from RGB images, applied to depth images to extract features. The intuition is to exploit the properties of guided filtering [52] i.e., spatially variant and content dependent for multi-modal fusion between RGB images and depth maps. However, this is computationally expensive; therefore, it proposes a convolution factorization operation to reduce computation and memory consumption.

Inspired by GuideNet [51], another method has been proposed, which aims to learn steering kernels [53] from RGB images and apply them to sparse depth maps to generate interpolated depth maps [54]. The interpolated depth maps are then refined by utilizing a ResNet [55] to generate the final dense depth maps. The whole pipeline can be trained in an end-to-end manner.

#### 2.2.2. Spatial Propagation Networks (SPN)

The aim of SPN is to learn an affinity matrix to represent the affinities between the pixels. An affinity matrix can be defined as a matrix containing the estimate of the likelihood that pixels (i and j) belong together conditioned on image measurements. The interpretation of the affinity matrix depends on the computer vision task. For instance, in the case of image segmentation task, the affinity matrix should contain semantic-level pairwise similarities.

Convolutional spatial propagation network (CSPN) [56] is one of the earliest methods, which proposed a generic framework for learning affinity matrix. Instead of manually designing an affinity matrix through similarity kernels for image segmentation, it learned semantic aware affinity values by utilizing deep convolutional neural network (CNN) [57]. Furthermore, the learned affinity matrix is not limited to single computer vision task, i.e., image segmentation [58], but it can also be extended to other vision tasks as well. However, it serially propagates the affinity matrix, making it inefficient for real-time applications.

CSPN [21] extended SPN and presented a convolutional network to learn the affinity matrix for the depth completion task. It argues that for a depth refinement task, affinity values of the local neighborhood are much more important [21]. To learn the affinity values in the local neighborhood, it utilized a deep convolutional neural network and to model long-range context, it uses a recurrent convolutional operation. However, both SPN and CSPN suffers from the problem of fixed local neighborhoods. To counter the problem of the fixed local neighborhood in CSPN and SPN, methods including CSPN++ [22], DSPN [59], NLSPN [11] and DySPN [60] were introduced.

CSPN++ [22] added a simple block to CSPN architecture to learn two additional hyper-parameters (1) adaptive convolutional kernel sizes, and (2) number of iterations for affinity matrix propagation based on image content. Initially, various configurations for both adaptive convolutional kernel sizes and the number of iterations for affinity matrix propagation are defined and then during propagation, it learns to predict the correct configuration on each pixel. This leads to significant improvement in both the runtime complexity and the accuracy of depth completion.

Unlike CSPN, DSPN [59] utilized deformable convolutional layers [61] to adaptively produce receptive field (kernels) and affinity matrix for each pixel. Later, NLSPN [11] was introduced, which utilized two-stage strategy for depth completion. In the first stage, the proposed method takes RGB and LiDAR sparse depth as an input and outputs (1) non-local neighbors and corresponding affinities of each pixel (2) initial depth estimate (3) confidence map of depth estimate. Then, in the second stage, non-local spatial propagation is iteratively performed with confidence-incorporated learnable affinity normalization to generate the final dense depth map. It counters the local affinity problem of CSPN through non-local spatial propagation.

Recently, DySPN [60] propose that instead of using linear propagation for generating affinity matrices, a non-linear propagation model should be used for propagation. It dynamically updates the pixel-wise affinity weights by utilizing neighborhood decoupling and spatial-sequential fusion. The neighborhood decoupling is performed by distributing the neighborhood based on the distances between a pixel and its neighborhood and then, recursively generating attention maps based on its propagation stage. Furthermore, it investigates three variants i.e., distance-based, dilated [45] and deformable convolutions for determining the optimal number of neighbors required for neighborhood decoupling. Finally, it proposes a diffusion suppression operation to reduce over smoothing of the predicted dense depth maps.

Another interesting use case of SPN is their utilization as a depth refinement networks. The original LiDAR sensor values are considered to be very accurate. However, the depth maps produced by the deep neural networks do not necessarily preserve the input depth values at valid pixels. Therefore, to recover the valid depth values, methods including PENet [32] and SemAttNet [35] utilize CSPN++ [22]. Furthermore, both SemAttNet and PENet incorporate dilated convolutions to enlarge and smoothen the transitions between the neighborhood. This further improves the propagation process and produces better results.

## 3. Multi-Modal Fusion

Multi-modal fusion refers to the approaches and methodologies of fusing sensor information from two or more different sensors to enhance the understanding of the environment. In the context of depth completion, it refers to the process of utilizing information from different modalities including RGB cameras [32,33], surface normal’s [20], semantic maps [34,35] etc., to guide the process of dense depth completion. The goal of multi-modal fusion is to leverage different modalities or their feature representations to produce reliable information on the sparse regions of LiDAR depth maps. Table 1 summarizes the fusion strategies along with their advantages and disadvantages.

### 3.1. Early Fusion

**Figure 3:**
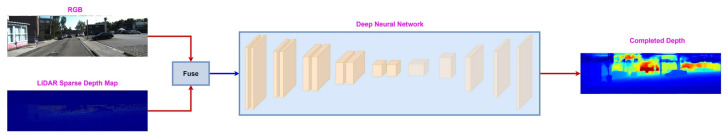
Early fusion between RGB image and LiDAR sparse depth. At first, both modalities are fused and then sent to the Deep Neural Network for dense depth completion.

The idea of early fusion is to integrate the separate raw modalities without any requirement of preprocessing e.g., RGB camera and LiDAR sensor, into a single unified representation [62] and encourage the learning of unimodal feature representations. Many methods exist to compute the joint representation of the multi-modal information. Most common methods include point pixel projection between RGB image and LiDAR sparse depth map [63], concatenation or addition of RGB and LiDAR sparse depth map [32,48], etc. The joint representation is then sent to a deep neural network for dense depth completion. The pipeline of early fusion is depicted in Figure 3.

### 3.2. Sequential Fusion

**Figure 4:**
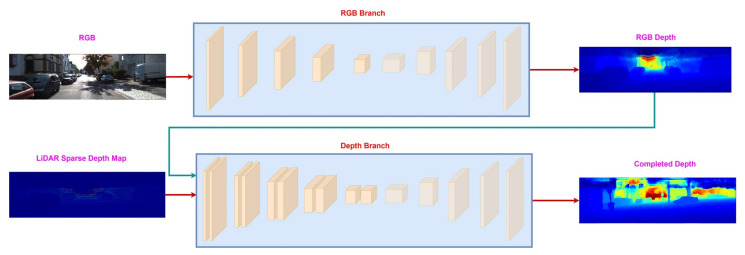
Sequential fusion between RGB image and LiDAR sparse depth map. The RGB branch produces color depth, which along with LiDAR sparse depth map, is sent to the depth branch to estimate the final dense depth map.

Sequential fusion is an extension of early fusion. The key idea is to solely predict the depth from RGB information and then use it to guide the depth-guided branch. Its a two-stage process, where, in the first step, it predicts a dense color depth through an RGB branch. Since the RGB branch doesn’t take any depth information as an input, the color depth is a very noisy estimate of dense depth. However, it contains the depth information around the object boundaries, e.g., cars and trees, which is missing in LiDAR sparse depth map [32,35,48]. In the second step, the color depth, and LiDAR sparse depth map are sent to the depth branch, which produces the final estimate of the dense depth map. Figure 4 shows the process of sequential fusion between RGB image and LiDAR sparse depth map.

**Table 1:** Comparison of fusion strategies.

Strategy	Key Idea	Advantages	Disadvantages
Early [20,22,23,32,33,35,48]	Creation of a unified representation of related input modalities e.g., RGB images and LiDAR sparse depth maps. The joint representation is passed as an input to a neural network for joint processing.	Outputs joint learned multi-modal feature representation. Single learning phase only for multi-modal information.	Loss of the information in creating joint representations. Synchronization between data modalities is required. Requirement of a method to create joint representations.
Sequential [30,32,35]	It is a multi-stage approach. The aim of the first stage is to focus on a single modality e.g., RGB image, and produce an intermediate output e.g., color depth, whereas, in the second stage, unimodal information including LiDAR sparse depth and color depth are combined to generate the final dense map.	No requirement of a method for creating joint representations of the multi-modal information.	Separate learning stage for each modality, which creates a learnable parameter overhead. Can be computationally expensive.
Late [17,32,35,48,51,64]	The idea is to process unimodal information (RGB, LiDAR) separately and then create a unified representation at the output level.	Targeted approaches to unimodal information can be applied as it focuses on the individual strength of modalities.	Does not focus on learning cross-correlations between the unimodal information.
Deep [33,35,48,51]	Performs fusion at intermediate (feature) level between the unimodal branches((RGB, LiDAR).	Primary focus is on learning cross-correlations between the unimodal branches. Active fusion at multiple locations and not just dependent on input/output.	Limited performance with naive feature fusion (addition, concatenation) methods.

### 3.3. Late Fusion

**Figure 5:**
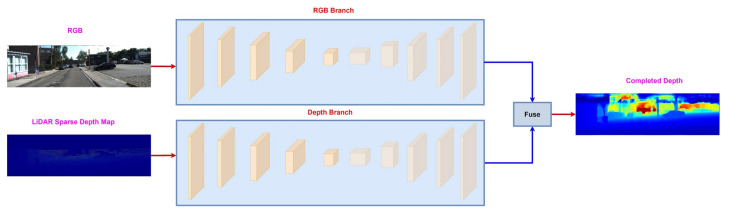
Late fusion between RGB image and LiDAR sparse depth map. It consists of two separate branches to process RGB images and LiDAR sparse depth maps. Both of the branches produce dense depth maps, which are fused to produce a final dense depth map.

Unlike early and sequential fusion, the late fusion processes both modalities, i.e., RGB color images and LiDAR sparse depth map, independently and fuses them at the final stage. The idea is to create a common representation, e.g., depth map from each branch, and then fuse them to create a unified output. The RGB and depth branches consist of RGB and depth-only deep neural networks. The RGB branch outputs a dense depth map focused on color information, whereas the depth branch produces a dense depth map relying more on the LiDAR sparse depth map features [32,35]. Since dense depth maps produced by RGB and depth branches are complementary, the final dense depth map combines the strength of both the RGB camera and LiDAR sensor into a single dense depth map. Figure 5 depicts the pipeline of the late fusion for the RGB camera and LiDAR sparse depth map.

### 3.4. Deep Fusion

**Figure 6:**
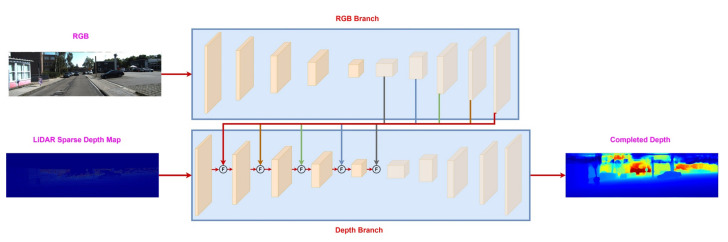
Deep Fusion between RGB image and LiDAR sparse depth map. Each modality is passed from a dedicated branch. The features from the decoder of the RGB branch are fused into the encoder of the depth branch. The symbol “F” represents the fusion operation. Common choices for fusion operation include addition or concatenation. However, complex fusion schemes can also be employed. By the guidance of the RGB branch, the depth branch produces a final dense depth map.

In contrast to earlier discussed fusion approaches, which apply fusion of modalities on the input or output, deep fusion is performed on the feature level of the sub-branches, thus enabling the exchange of information between the multi-modal information thought the network. Figure 6 shows the pipeline of the deep fusion between LiDAR sparse depth map and RGB image modalities. The pipeline of deep fusion consists of two separate branches for RGB and LiDAR sparse depth modalities. The fusion follows the decoder-encoder strategy since the features from the RGB decoder are fused at the encoder of the depth branch at multiple stages. It only fuses the decoder features of one modality to another because the decoder contains high-level information, which is used to guide the other modality during dense depth prediction [32,35,48,51].

## 4. Datasets

Typically, depth completion is applied to two kinds of datasets i.e., outdoor and indoor datasets. The outdoor datasets consist of driving sequences, whereas indoor datasets comprise video sequences from a variety of indoor scenes. There exist many such datasets; however, in this paper, we will discuss two famous datasets and benchmarks i.e., KITTI Dataset and its Depth Completion Benchmark (outdoor) [65] and NYU Depth Dataset v2 (indoor) [66], which are used extensively in the field of depth completion for evaluation. The following sections will discuss both KITTI and NYU-v2 datasets in detail.

### 4.1. KITTI Dataset

KITTI dataset [65] is a large outdoor dataset for autonomous vehicles comprising driving sequences recorded in Karlsruhe, Germany. The driving vehicle VW Passet station is equipped with two stereo camera systems, a LiDAR Velodyne HDL-64E laser scanner, and an OXTS RT3003 inertial and GPS navigation system. Most of the scenes are collected in rural areas and on the city’s highways, which sum up-to hours of various driving scenarios. Furthermore, the KITTI dataset provide various benchmarks on different challenging tasks such as 2D/3D object detection, depth map completion, semantic segmentation, and tracking. However, in this paper, we will only focus on reviewing the techniques associated with the LiDAR sparse depth completion benchmark.

#### KITTI Depth Completion Benchmark

**Figure 7:**
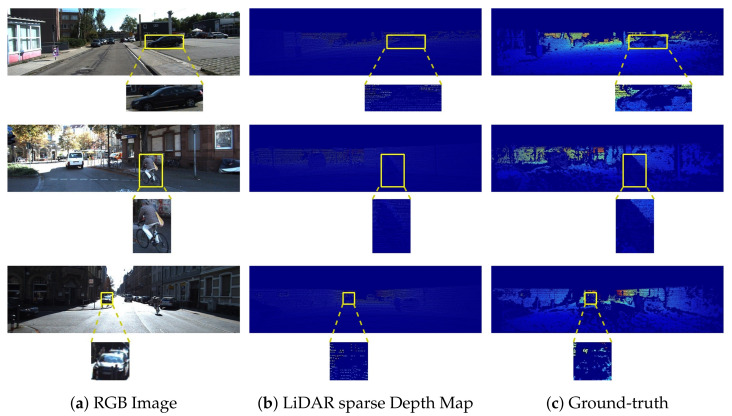
KITTI depth completion benchmark. Part (**a**) shows the aligned RGB images. Part (**b**) depicts the sparse LiDAR depth maps, whereas Part (**c**) represents the dense ground-truth depth maps. Colorization is applied on LiDAR sparse depth maps and corresponding ground-truth to generate visualizations. The highlighted areas are used to show the sparsity in KITTI depth completion benchmark.

KITTI depth completion [18] benchmark is one of several benchmarks, which are provided by KITTI [65]. It is a very famous benchmark and consists of over 100 entries on its official online leaderboard. It contains 850,000 LiDAR sparse depth maps with aligned RGB images for training, 7000 for validation, and 1000 for testing of methods. Velodyne’s HDL-64E LiDAR sensor is used to generate the depth maps of the scene, whereas RGB images are captured through pair of stereo cameras. Due to limited resolution and scan lines, the LiDAR sensor provide valid depth values on only 5.9% of all pixels [18,65]. Furthermore, the corresponding ground-truth provided by KITTI depth completion benchmark contains valid depth values on 16% of all the pixels. The ground-truth is dense, since it is computed by accumulating LiDAR and stereo estimation of the scenes through semi-global matching (SGM) [67] approach. Furthermore, the KITTI depth completion dataset also provides an official validation set consisting of 1000 frames. Figure 7 presents some images from the depth completion benchmark.

### 4.2. Nyu-v2 Depth Dataset

**Figure 8:**
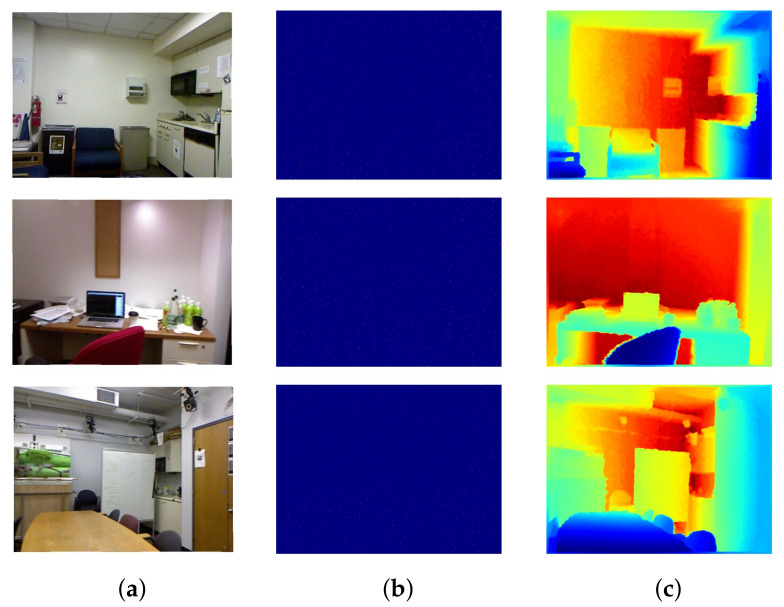
Nyu-v2 depth dataset. Part (**a**) shows the aligned RGB images. Part (**b**) depicts the sparse Kinect depth maps, which are generated by randomly sampling only 500 points from the ground truth. Part (**c**) represents the fully dense ground-truth depth maps. Colorization is applied on Kinect sparse depth maps and corresponding ground-truth to generate visualizations. (**a**) RGB Image. (**b**) Kinect sparse Depth Map. (**c**) Ground-truth.

It consists of RGB and depth images collected from 464 different indoor scenes. It utilizes a camera to capture RGB data and Microsoft Kinect [68] to record the depth values of the scene. As a preprocessing step, the missing values in depth maps are colorized using a colorized scheme [69]. It provides over 400K images for training; however, most of the methods [32,33,40,51] utilize only a subset for training their approaches. As Kinect provide dense measurements [68], the sparse depth data is generated by randomly removing depth data from the depth ground truth. It also provides 654 images for benchmarking of the results. Figure 8 shows some images from the Nyu-v2 depth dataset.

## 5. Evaluation Metrics

The most common depth completion evaluation metrics are defined as follows.

(1) $$\begin{array}{cc} Root\;Mean\;Squared\;Error(\mathrm{mm}) & =\sqrt{\frac{1}{\left|V\right|}\sum_{v\in V}{\left|d_{v}^{gt}-d_{v}^{pred}\right|}^{2}} \end{array}$$

(2) $$\begin{array}{cc} Mean\;Absolute\;Error(\mathrm{mm}) & =\frac{1}{\left|V\right|}\sum_{v\in V}\left|d_{v}^{gt}-d_{v}^{pred}\right| \end{array}$$

(3) $$\begin{array}{cc} Root\;Mean\;Squared\;Error\;of\;Inverse\;Depth(\frac{1}{\mathrm{km}}) & =\sqrt{\frac{1}{\left|V\right|}\sum_{v\in V}{\left|1/d_{v}^{gt}-1/d_{v}^{pred}\right|}^{2}} \end{array}$$

(4) $$\begin{array}{cc} Mean\;Absolute\;Error\;of\;Inverse\;Depth(\frac{1}{\mathrm{km}}) & =\frac{1}{\left|V\right|}\sum_{v\in V}\left|1/d_{v}^{gt}-1/d_{v}^{pred}\right| \end{array}$$

(5) $$\begin{array}{cc} Mean\;Absolute\;Relative\;Error\;Depth(\mathrm{mm}) & =\frac{1}{V}\sum_{i=1}^{v}\frac{\left|d_{i}^{pred}-d_{i}^{gt}\right|}{d_{i}^{gt}} \end{array}$$

(6) $$\begin{array}{cc} Threshold\;Accuracy(\delta ) & =\max\left(\frac{d_{i}^{pred}}{d_{i}^{gt}},\frac{d_{i}^{gt}}{d_{i}^{pred}}\right)=\delta <\tau \end{array}$$

where $d_{v}^{gt}$ represents ground-truth, $d_{v}^{pred}$ depicts predicted depth map and τ represents the threshold.

Among all of the evaluation metrics, RMSE is chosen to rank the submissions on the KITTI and Nyu-v2 Depth online leaderboards.

## 6. Objective Functions

In the field of depth completion, the design of an objective function is critical to the success of the approach. Since there exists both supervised [20,32,33,35] and unsupervised [15,70,71,72] methods to depth completion problem, objective functions can be categorized based on the choice of learning strategy. The common loss functions for each strategy is defined below.

### 6.1. Supervised Learning

Given a LiDAR sparse depth map $d^{sd}$, the predicted dense depth map $d^{pred}$ and the ground truth $d^{gt}$, various existing methods [39,48,60] utilize $\ell_{1}$ norm as a loss function between $d^{sd}$ and $d^{pred}$. It is defined as follows

(7) $$\begin{array}{cc} \ell_{1} & =\frac{1}{\left|n\right|}\sum_{i\in n}{∥d_{i}^{gt}-d_{i}^{pred}∥}_{1} \end{array}$$

where $\left|\right|d_{i}^{gt}-d_{i}^{pred}{\left|\right|}_{1}$ defines the $\ell_{1}$ norm between the predicted depth values and ground truth. However, $\ell_{1}$ norm gives the same weight to each pixel irrespective of its location. This is only sub-optimal since depth completion is considered more difficult and challenging at the farthest points.

To counter this limitation, many methods [20,32,33,35,43] utilize $\ell_{2}$ norm. The $\ell_{2}$ norm is more sensitive to outliers and penalizes the points on further distance. The $\ell_{2}$ norm between $d^{sd}$ and $d^{pred}$ is given as follows.

(8) $$\begin{array}{cc} \ell_{2} & =\frac{1}{\left|n\right|}\sum_{i\in n}{∥d_{i}^{gt}-d_{i}^{pred}∥}_{2} \end{array}$$

where $\ell_{2}$ norm between the predicted depth values and ground truth is depicted by $\left|\right|d_{i}^{gt}-d_{i}^{pred}{\left|\right|}_{2}$. Both $\ell_{1}$ and $\ell_{2}$ norms are calculated with direct supervision.

Along with the norm-based loss functions, many works [34,43,71] utilize structural similarity index measure (SSIM) [73] to constrain the luminance, contrast, and structure of the predicted dense depth maps.

### 6.2. Unsupervised Learning

For unsupervised learning, proposed approaches focus on learning smoothness [15,70] and photometric loss [15,72] functions. Photometric loss can be used to generate a supervisory signal for the depth completion problem. The idea of photometric loss is to exploit the temporal information and warp the predicted dense depth map to a nearby color image. Furthermore, the pixel differences between the warped image (RGB) and nearby color image compute the respective error. Mathematically, the photometric loss is defined as follows.

(9) $$\begin{array}{c} L_{\mathrm{photometric}\;}\left(\mathrm{warped},\mathrm{RGB}\right)=\sum_{i\in n}\frac{1}{n}{∥{𝟙}_{\left\{d=0\right\}}^{\left(i\right)}\cdot \left({\mathrm{warped}}^{\left(i\right)}\;-\;{\mathrm{RGB}}^{\left(i\right)}\right)∥}_{1} \end{array}$$

Unlike supervised learning-based objective functions, the photometric loss is only determined where the ground truth is not available.

Since photometric loss only focuses on the sum of individual pixel error values [15], it encourages discontinuity in the local neighborhood of the pixels. The discontinuity can result in high error values in the predicted dense depth maps [15,70]. To overcome this problem, a smoothness term is added to the objective function, which ensures the smoothness of depth predictions. It is applied by computing second-order gradients of predicted dense depth maps as shown in the equation given below.

(10) $$\begin{array}{c} L_{\mathrm{smooth}}\left(d_{pred}\right)=\frac{1}{n}\sum_{i=1}^{n}\left(\left|\frac{\partial_{x}^{2}}{\partial x^{2}}\cdot d_{pred}^{i}\right|+\left|\frac{\partial_{y}^{2}}{\partial y^{2}}\cdot d_{pred}^{i}\right|\right) \end{array}$$

## 7. Results

**Figure 9:**
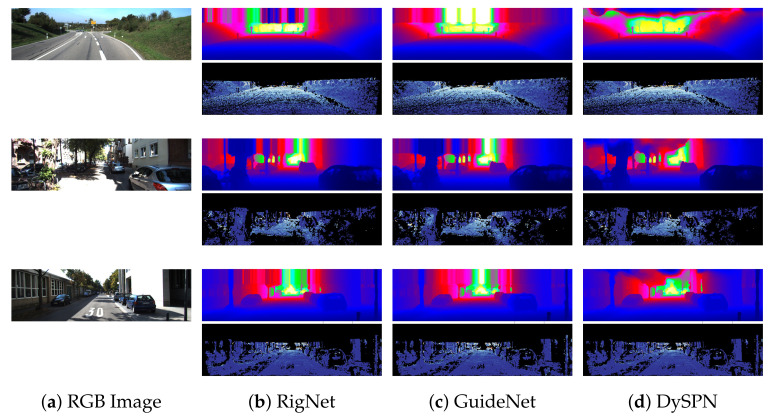
Qualitative comparison of the top three reported methods on KITTI depth completion test set, including (**b**) RigNet [33], (**c**) GuideNet [51], and (**d**) DySPN [60]. Given sparse depth maps and the input guidance color images (**a**), the methods output dense depth predictions (1st row). The corresponding error maps (2nd row) are taken from the KITTI leaderboard for comparison. Warmer color represents higher error.

This section compares the results from all the state-of-the-art approaches reviewed above. The performance comparison is made both quantitatively and qualitatively. The quantitative results are reported on the two benchmark datasets for depth completion i.e., KITTI autonomous driving scenes dataset and the NYUv2 indoor scenes dataset. The results on the KITTI dataset are evaluated using the four standard metrics; root mean squared error (RMSE), mean absolute error (MAE), root mean squared error of the inverse depth (iRMSE), and mean absolute error of the inverse depth (iMAE) as shown in Table 2. For the indoor NYUv2 dataset, three metrics are used for evaluation, including the RMSE, mean absolute relative error (REL), and $\delta_{i}$. Table 3 shows the performance results on the NYUv2 indoor scenes dataset. Qualitative results for the top performing technique from each category are presented in Figure 9. Since there is no public leaderboard for the NYUv2 Benchmark dataset, therefore, we have not added their qualitative results.

Unguided approaches try to directly achieve dense depth maps from sparse depth maps, which causes discontinuities in depth values and loss of structural information. Modern image-guided approaches outperform the unguided ones by a fair margin by using an image as guidance. Spatial propagation methods learn affinity matrices and propagate these to make depth denser. DySPN [60] is the most successful technique in this category and uses non-linear propagation resulting in smoother depth maps. Among the multi-branch image-guided approaches, RigNet [33] achieves the best results on both the KITTI [65] and NYUv2 [66] datasets. Lastly, GuideNet [51] is the most noticeable work under the guided image filtering category. Overall, we conclude that image-guided multi-branch networks show the best results and are currently the state-of-the-art in depth completion. The proper use of multi-modality data allows for the resolution of blurry guidance in images and unclear structure in depth. Also, multi-scale fusion techniques employed by some of the multi-branch methods [32,48] prove most successful in extracting discriminate features and fusing them with sparse depth data.

**Table 2:** Comparison of State-of-the-art approaches on the KITTI Benchmark test dataset. The methods are ordered by their RMSE results from worst to best within each category. The best results within each category are mentioned in bold letters.

Category	Method	RMSE	MAE	iRMSE	iMAE
Multi-branch Networks	SSGP [47]	838.00	245.00	-	-
	DDP [39]	836.00	205.40	2.12	0.86
	MS-Net[LF]-L2 [19]	829.98	233.26	2.60	1.03
	S2D [15]	814.73	249.95	2.81	1.21
	CrossGuidance [44]	807.42	253.98	2.73	1.33
	RSIC [46]	792.80	225.81	2.42	0.99
	Depth-normal [38]	777.05	235.17	2.42	1.13
	FusionNet [23]	772.87	215.02	2.19	0.93
	MSG-CHN [41]	762.19	220.41	2.30	0.98
	DeepLiDAR [20]	758.38	226.50	2.56	1.15
	DenseLiDAR [43]	755.41	214.13	2.25	0.96
	ACMNet [40]	744.91	206.09	2.08	0.90
	FCFR-Net [48]	735.81	217.15	2.20	0.98
	PENet [32]	730.08	210.55	2.17	0.94
	**RigNet** [33]	**712.66**	203.25	2.08	0.90
Guided Image Filtering	GuideNet [51]	739.24	218.83	2.25	0.99
Spatial Propagation Networks	CSPN [21]	1019.64	279.46	2.93	1.15
	DSPN [59]	766.74	220.36	2.47	1.03
	CSPN++ [22]	743.69	209.28	2.07	0.90
	NLSPN [11]	741.68	199.59	1.99	0.84
	**DySPN** [60]	**709.12**	192.71	1.88	0.82

**Table 3:** Comparison of state-of-the-art approaches on the NYUv2 Benchmark dataset. Performances are reported for 500 samples. The methods are ordered by their RMSE results from worst to best within each category. The best results within each category are mentioned in bold letters. $\delta_{i}$ denotes the percentage of predicted pixels whose relative error is less than a threshold *i* (1.25, 1.25${}^{2}$, and 1.25${}^{3}$).

Category	Method	RMSE	REL	$\delta_{1.25}$	$\delta_{1.25^{2}}$	$\delta_{1.25^{3}}$
Multi-Branch Networks	S2D [15]	0.133	0.027	-	-	-
	EncDec-Net[EF] [19]	0.123	0.017	99.1	99.8	100
	DeepLiDAR [20]	0.115	0.022	99.3	99.9	100.0
	Xu et. al. [38]	0.112	0.018	99.5	99.9	100.0
	FCFR-Net [48]	0.106	0.015	99.5	99.9	100.0
	ACMNet [40]	0.105	0.015	99.4	99.9	100
	DenseLiDAR [43]	0.105	0.015	99.4	99.9	100
	**RigNet** [33]	**0.090**	0.013	99.6	99.9	100.0
Guided Image Filtering	GuideNet [51]	0.101	0.015	99.5	99.9	100.0
Spatial Propagation Networks	CSPN [21]	0.117	0.016	99.2	99.9	100.0
	CSPN++ [22]	0.116	-	-	-	-
	NLSPN [11]	0.092	0.012	99.6	99.9	100.0
	**DySPN** [60]	**0.091**	0.012	99.6	99.9	100.0

## 8. Conclusions

In this paper, we present a comprehensive survey of depth completion methods. We first present a basic hierarchy of depth completion methodologies consisting of Unguided and Image-guided methods. The Image-guided approaches are subdivided into Multi-branch and Spatial propagation networks. The Multi-branch networks further contain a special branch of methods classified as Guided Image Filtering methods. Then, we review the different state-of-the-art approaches within each category of the hierarchy by summarizing their contributions and their approach to resolving the prevalent problems of the domain. We further shed light on the most popular benchmark datasets among the research fraternity and the corresponding evaluation metrics reported on each. Finally, to give an overall picture, we present a comparison of all the methods on the discussed benchmarks and reported metrics and concisely mention their pros and cons.

## Data Availability

Not applicable.
